# Supplementary material for: Plasmonic high-entropy carbides
Source: Nat Commun. 2022 Oct 11;13:5993. doi: 10.1038/s41467-022-33497-1 (PMC9553889; doi:10.1038/s41467-022-33497-1)
Supplement: Supplementary file 1 — Supplementary Information [file 41467_2022_33497_MOESM1_ESM.pdf]

Supplementary Information
Plasmonic high-entropy carbides

Arrigo Calzolari,[1,*] Corey Oses,[2,3] Cormac Toher,[4,3] Marco Esters,[2,3]
Xiomara Campilongo,[3] Sergei P. Stepanoff,[5] Douglas E. Wolfe,[5] and Stefano Curtarolo[2,3,†]

[1] *CNR-NANO Research Center S3, Via Campi 213/a, 41125 Modena, Italy*
[2] *Department of Mechanical Engineering and Materials Science, Duke University, Durham NC, 27708 USA*
[3] *Center for Autonomous Materials Design, Duke University, Durham, NC 27708, USA*
[4] *Department of Materials Science and Engineering and Department of Chemistry and Biochemistry, University of Texas at Dallas, Richardson, TX 75080, USA*
[5] *Applied Research Laboratory, The Pennsylvania State University, University Park, PA 16802, USA*

(Dated: September 20, 2022)

* arrigo.calzolari@nano.cnr.it
† stefano@duke.edu

SUPPLEMENTARY NOTES

Supplementary Note 1. Transition metals carbides

Supplementary Figure 1 shows the Electron Energy Loss Spectra (EELS, straight green line) and reflectivity (dashed blue line) the optical properties of parent transition metal carbides (TMCs) in the rocksalt fcc crystalline phase. At normal incident conditions, the reflectivity (R) is defined as $R = \frac{(1-n)^2+k^2}{(1+n)^2+k^2}$, where $n = \{\frac{1}{2}[(\epsilon_1^2 + \epsilon_2^2)^{1/2} + \epsilon_1]\}^{1/2}$ and $k = \{\frac{1}{2}[(\epsilon_1^2 + \epsilon_2^2)^{1/2} - \epsilon_1]\}^{1/2}$ are the real and imaginary part of the complex refractive index. In ideal Drude metals $R = 1$ for $E < E_0$ and $R = 0$ for $E > E_0$. The differences of realistic plasmonic high-entropy carbides (PHECs) with respect to the ideal case are associated to energy loss dissipation due to interband optical transitions. Only TaC exhibit a remarkable plasmonic resonance in the visible range.

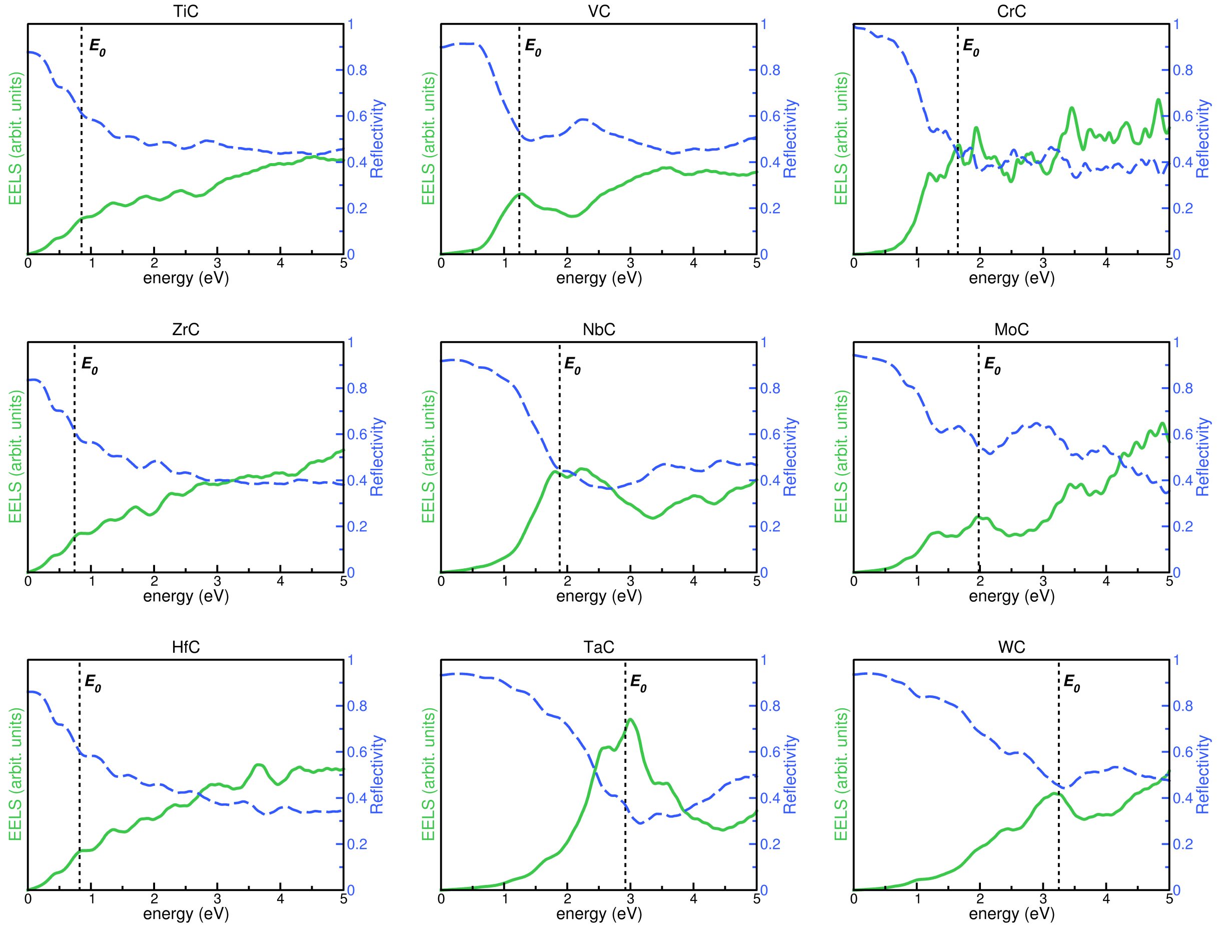

Supplementary Figure 1. Electron Energy Loss Spectra (straight green line) and reflectivity (dashed blue line) of transition metal carbides. Source data are provided as a Source Data file.

The electronic bandstructure plots and the corresponding total and projected density of states of TMCs are summarized in Supplementary Figure 2.

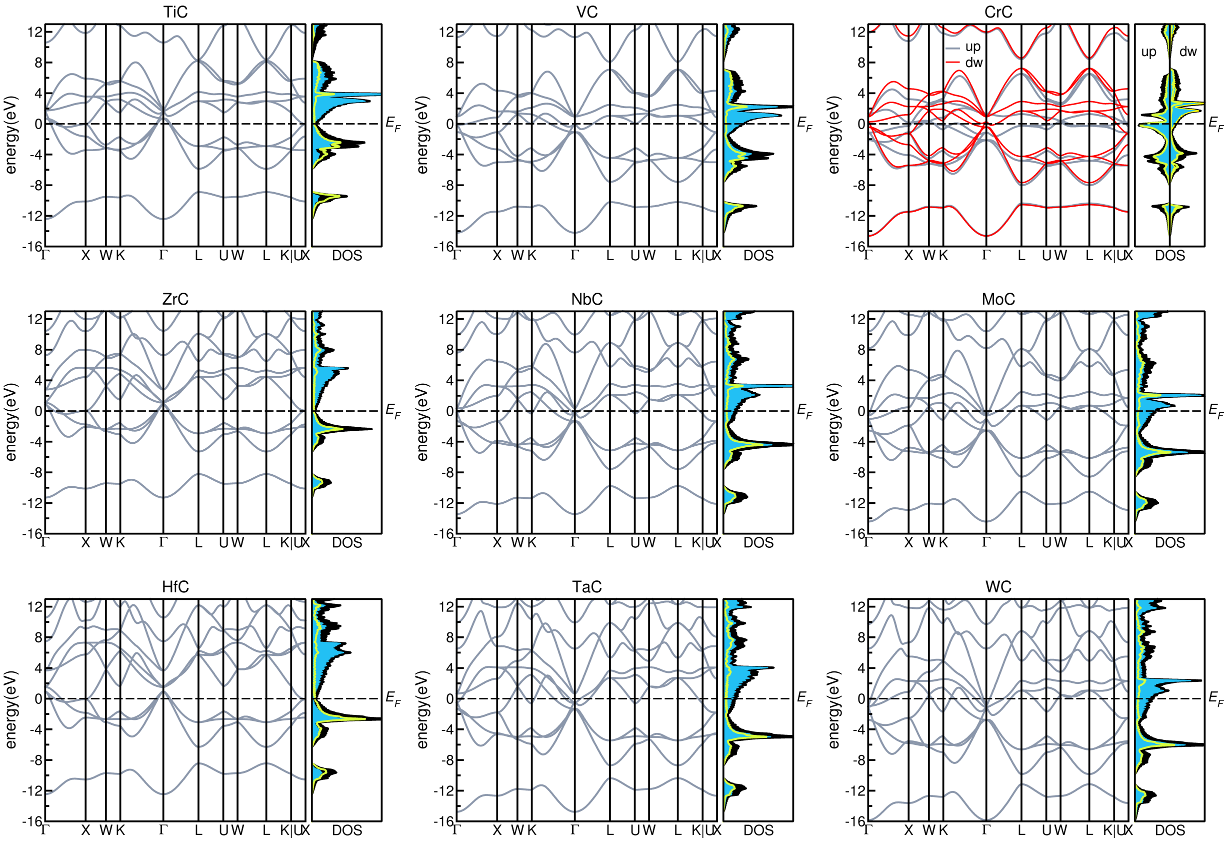

Supplementary Figure 2. Calculated bandstructure (left side) and projected electronic density of states (eDOS) (right side) of transition metal carbides. Zero energy reference is set to the Fermi energy (dashed line) of each system. On left sides, the black area corresponds to the total eDOS, while the cyan area and green line correspond to TM- and C-projected eDOS. Source data are provided as a Source Data file.

Supplementary Note 2. Simulation of optical properties using POCC

Here we test the capability of the POCC method in simulating the optical spectra of disordered systems. We consider the case of the disordered AuAg solid-solution, compared to the Au and Ag fcc constituents, all well-known plasmonic systems. AuAg is simulated by using 44 POCC structures with 8 atoms per cell. The imaginary part ϵ_i of the dielectric function of the three systems is shown in Supplementary Figure 3(a), along with the corresponding experimental counterpart (inset), extracted from Ref. [1]. The disordered alloy has a distinct character, with spectral features not directly ascribable to the ordered crystalline phases of the single elements. The excellent agreement with the experimental findings confirms the accuracy of the present approach in including the effect of disorder into the description of the optical properties.

The excitation of plasmons is a purely electronic effect, i.e., intraband electron-electron excitations. The excitation of the electron gas is an ultrafast process (on the femtosecond scale) that does not involve the interaction with phonons. The ionic temperature has an indirect and minor effect of the plasmon excitation energies, mostly ascribable to the thermal expansion of the lattice and thus to the average modifications of the bond lengths. Supplementary Figure 3(b) shows the simulated EELS spectra of disordered AuAg solid-solution at different configurational temperatures. The results match well with the measured redshift of the plasmonic band (see gray dashed line) of AuAg as the temperature is increased [2]. This effect, usually of the order of 0.1 eV, has been observed also for Ag bulk systems and it is associated to thermal structural expansion [3]. As shown in Supplementary Figure 1(b), this effect is reproduced by POCC, which describes the spatial distribution of the atoms in the disordered system. Beyond a certain temperature (system dependent), structural instabilities and/or phase transitions (e.g., melting), will change the morphology of the system, yet affecting optical properties. This is not considered in the article because it focuses on thermodynamically homogeneous systems. Therefore, we can conclude that away from critical temperatures, temperature has a secondary

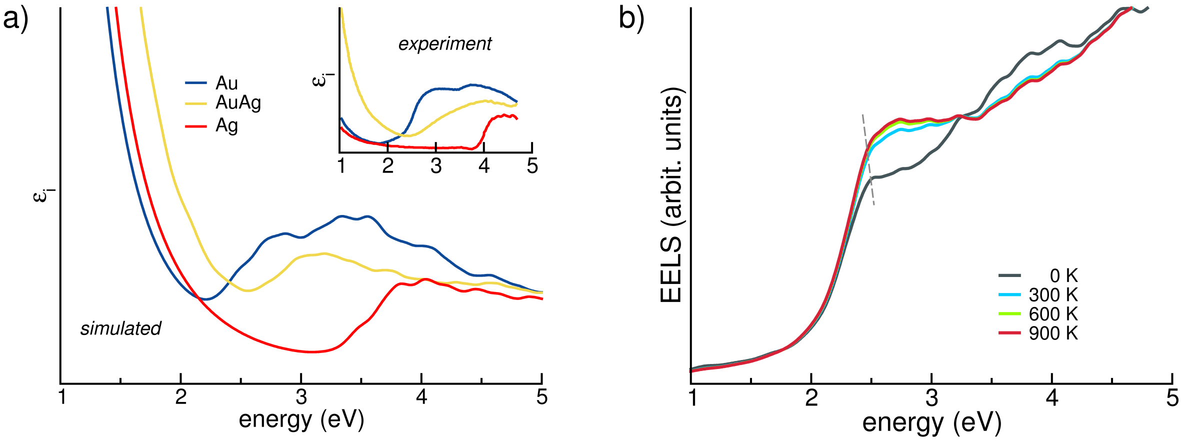

Supplementary Figure 3. **(a)** Imaginary part (ϵ_i) of the complex dielectric function of Au (blue), Ag (red) and AuAg (yellow) systems. Inset reports the corresponding experimental spectra as extracted from Figure 3(b) of Ref. [1]. **(b)** Simulated Electron Energy Loss spectrum (EELS) of disordered AuAg solid-solution at different configurational temperature. Source data are provided as a Source Data file.

effect on the plasmonic excitation energy and that our approach well describes this process.

The stability (i.e., lifetime) and of the de-excitation of the plasmonic resonance follows a different scenario [4, 5]. Here, the interaction with the lattice, and thus with the ionic temperature, is critical. After the initial ultrafast thermalization of the hot-electron gas through electron-electron scattering, the energy is transferred to the lattice via phonon emission (electron–phonon coupling) on the nanosecond scale. Finally, thermal energy is radiated (heat-diffusion) towards the external environment through phonon-phonon scattering on the micro-to-millisecond scale. While increasing temperature may favor the plasmon extinction through destructive electron-phonon interaction, it does not directly affect the energy position of the plasmon resonance and/or of the crossover energy (beyond considerations about thermal expansion). Therefore, we can conclude that below the critical melting point, temperature has a minor effect on the plasmonic excitation energy and that the POCC method well characterizes the phenomenon.

Supplementary Note 3. Chemical modifications in $HfTa_4C_5$

Starting from optimized $HfTa_4C_5$, we calculated the optical properties of derived materials. Simulations on these derived materials have been done on a single POCC structure and without further atomic relaxation. Supplementary Figure 4 shows the EELS spectra for $Hf_xTa_{5-x}C_5$ model, by varying the amount of Hf in the cell, with $x = [0-5]$. The cases $x = 0$ and $x = 5$ correspond to TaC and HfC, respectively. Supplementary Figure 5 shows the EELS spectra for $M Ta_4C_5$ model, with M=Ti, Zr, Hf, V, Nb, Cr, Mo, and W.

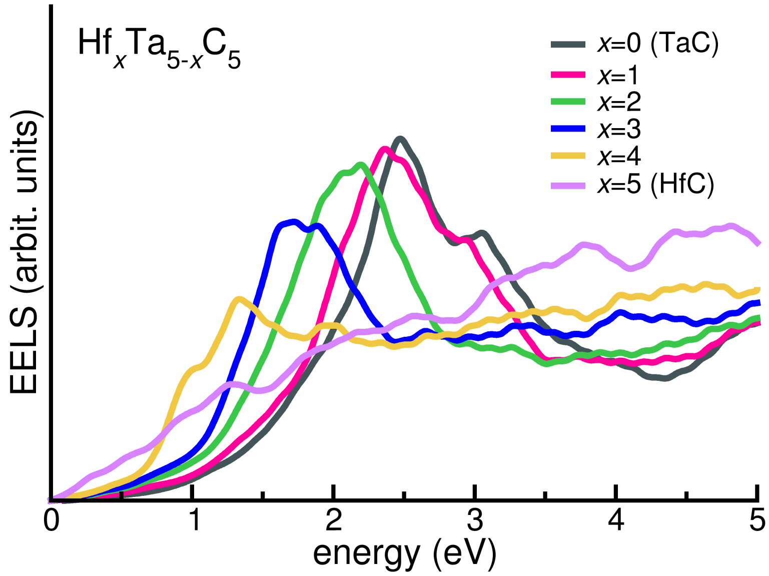

Supplementary Figure 4. Simulated EELS spectra $Hf_xTa_{5-x}C_5$ model, with $x = [0-5]$. Source data are provided as a Source Data file.

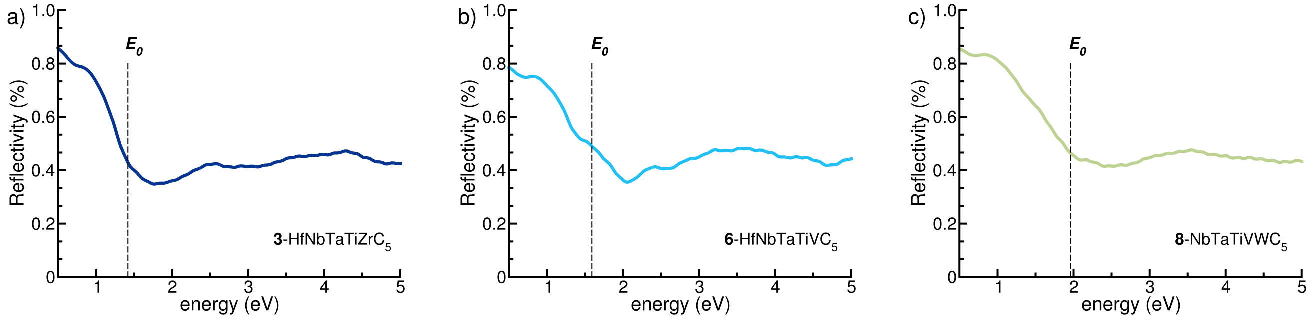

Supplementary Figure 5. Simulated EELS spectra in the low energy range for the $M Ta_4C_5$ model with **(a)** M=Hf, Ti, Zr, **(b)** M=Hf, V, Nb, and **(c)** M=Hf, Cr, Mo, W. Source data are provided as a Source Data file.

Supplementary Figure 6 shows the dielectric function and the EELS spectra of crystalline HfC rocksalt structure, included for comparison.

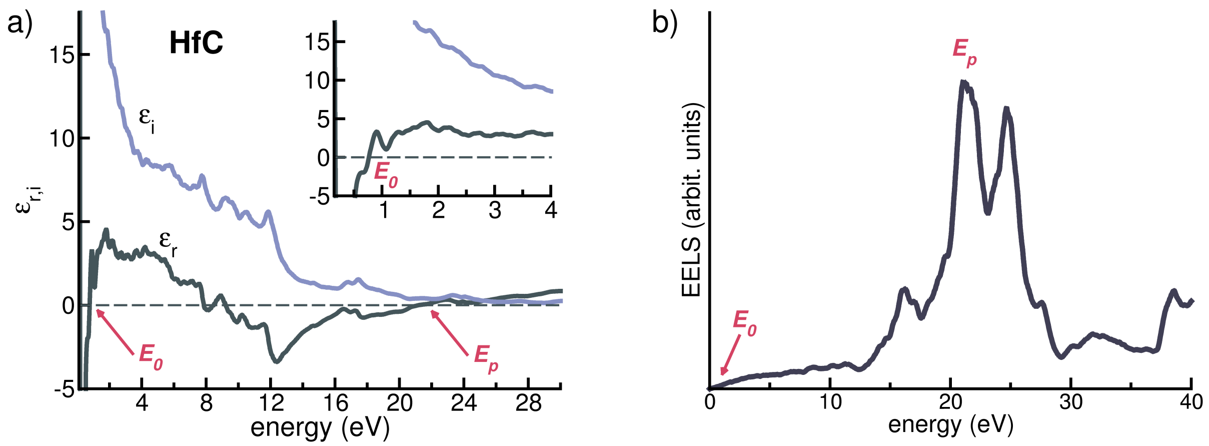

Supplementary Figure 6. Optical properties of HfC fcc rocksalt crystal. **(a)** Real and imaginary part of the dielectric function (inset zooms on the lowest energy part of the spectrum). **(b)** Simulated EELS spectra. Source data are provided as a Source Data file.

Supplementary Note 4. Plasmonic High-Entropy Carbides

Simulated reflectivity spectra for selected PHECs.

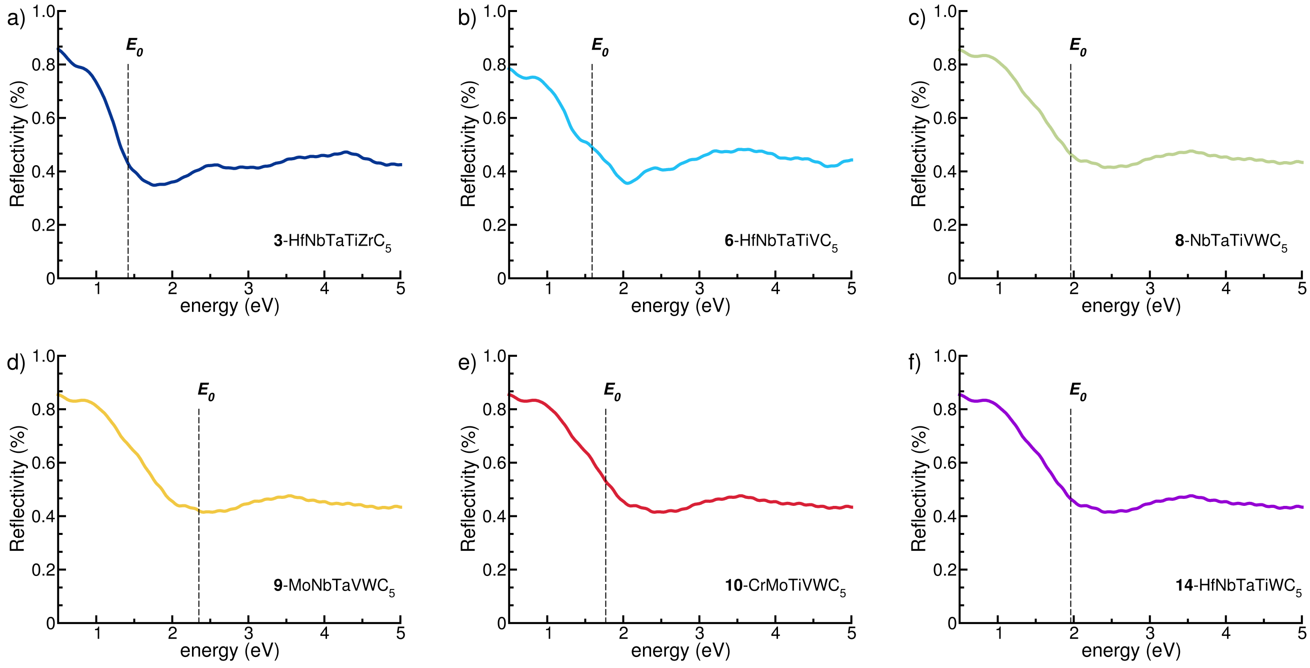

Supplementary Figure 7. Simulated reflectivity spectra for selected PHECs. E_0 indicates the crossover energy. **(a)** **3**-HfNbTaTiZrC$_5$; **(b)** **6**-HfNbTaTiVC$_5$; **(c)** **8**-NbTaTiVWC$_5$; **(d)** **9**-MoNbTaVWC$_5$; **(e)** **10**-CrMoTiVWC$_5$; **(f)** **14**-HfNbTaTiWC$_5$. Source data are provided as a Source Data file.

Supplementary Note 5. Single particle vs TDDFT approach

Supplementary Figure 8 shows the comparison between the complex dielectric function of (a) fcc TaC rocksalt crystal and (b) **3**-HfNbTaTiZrC_5 high entropy carbide, calculated with Time-Dependent Density Functional Perturbation Theory (TD-DFPT) model used in this work [6], and with the single particle (SP) approach based on the Drude-Lorentz in the limit for transferred momentum $\mathbf{q} \rightarrow \mathbf{0}$. Both approaches are implemented in the Quantum ESPRESSO distribution [7]. For both systems, the two spectra are identical, within the numerical errors.

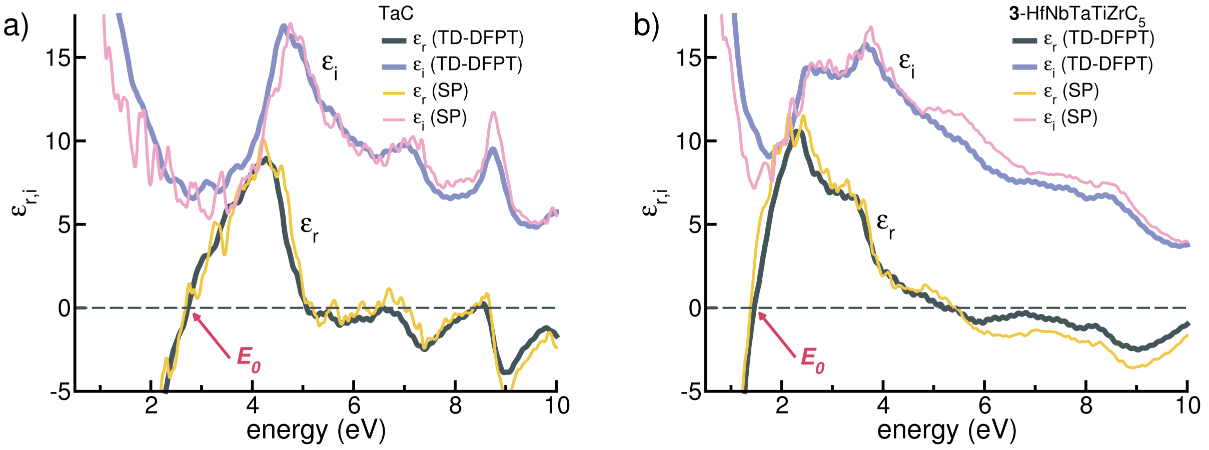

Supplementary Figure 8. Comparison between the optical complex dielectric function $\hat{\epsilon}(E) \equiv (\epsilon_r + i\epsilon_i)$ calculated with the Liouville-Lanczos TD-DFPT approach and the Drude-Lorentz single particle (SP) approach, as implemented in the Quantum ESPRESSO suite of codes for **(a)** fcc TaC rocksalt crystal, and **(b)** **3**-HfNbTaTiZrC_5 high entropy carbide (single pocc). ϵ_r, ϵ_i, and E_0 identify the real, (imaginary) part of the dielectric function and the crossover energy, respectively. Source data are provided as a Source Data file.

Supplementary Note 6. Experimental measurements

Electron transparent lamellae were prepared for EELS analysis from the center of a cross-sectioned $HfTa_4C_5$ pellet in a Thermofisher Scientific Helios NanoLab 660 Dual Beam focused ion beam (FIB)-scanning electron microscope (SEM) using a 30 keV Ga ion beam. A sacrificial carbon protective layer was deposited on top of the $HfTa_4C_5$ prior to milling to limit excess ion implantation as the cross section was thinned to electron transparency, after which each face of the lamellae was polished using a 5 keV Ga ion beam to remove resputtered material and reduce the thickness of any amorphous implantation layer produced during milling at high accelerating voltages. Once thinned, the sections were lifted and transported to a Protochip Fusion Select Heating E-chip and micro-welded to the chip using localized 5 keV ion assisted deposition of carbon. Supplementary Figure 9 shows the thinned and polished cross section after transport to the heating chip. Panel (b) shows a monochromated STEM image of the $HfTa_4C_5$ lamellae, which indicates the region where all EELS spectra were collected as a function of temperature.

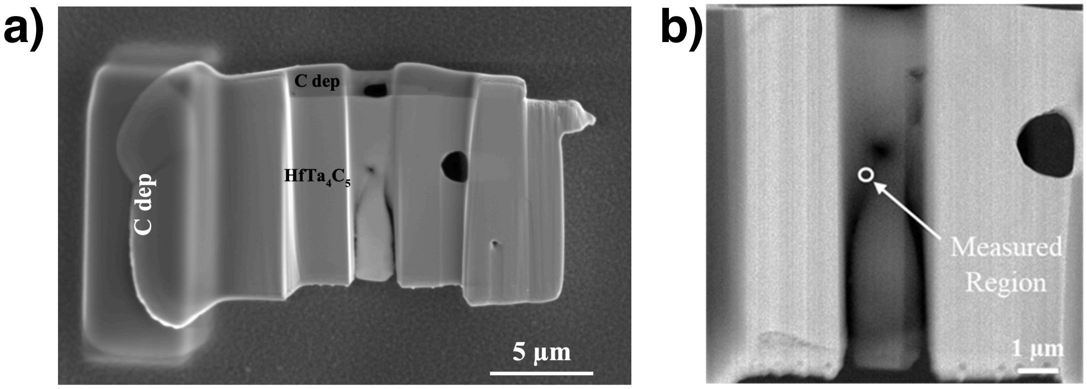

Supplementary Figure 9. **(a)** Ion milled electron transparent cross-section of sintered $HfTa_4C_5$ pellet fixed to Protochip Fusion Select Heating E-chip. The center of the cross-section was thinned to electron transparency and polished using a Ga ion beam prior to transfer to the E-chip, where the section was micro-welded to the chip using Ga ion assisted deposition of carbon as indicated on the left of the figure. **(b)** Monochromated STEM micrograph of the $HfTa_4C_5$ cross-section, which indicates the region where all EELS spectra were collected. Source data are provided as a Source Data file.

SUPPLEMENTARY REFERENCES

[1] D. Rioux, S. Vallieres, S. Besner, P. Munoz, E. Mazur, and M. Meunier, *An Analytic Model for the Dielectric Function of Au, Ag, and their Alloys*, Adv. Opt. Mater. **2**, 176–182 (2014).

[2] S. Pramanik, S. Chattopadhyay, J. K. Das, U. Manju, and G. De, *Extremely fast Au-Ag alloy-dealloy associated reversible plasmonic modifications in SiO_2 films*, J. Mater. Chem. C **4**, 3571–3580 (2016).

[3] U. Kreibig and M. Vollmer, *Optical Properties of Metal Clusters*, Springer Series in Materials Science p. 317 (1995).

[4] R. Rosei, F. Antongeli, and U. M. Grassano, *d-bands position and width in gold from very low temperature thermomodulation measurements*, Surf. Sci. **37**, 689–699 (1973).

[5] Aeschlimann, M. Electron Dynamics in Metallic Nanoparticles in *Encyclopedia of Nanoscience and Nanotechnology*, Ed. by Nalwa N. **2004** American Scientific Publishers, USA.

[6] I. Timrov, N. Vast, R. Gebauer, and S. Baroni, *Electron energy loss and inelastic x-ray scattering cross sections from time-dependent density-functional perturbation theory*, Phys. Rev. Lett. **88**, 064301 (2013).

[7] P. Giannozzi, O. Andreussi, T. Brumme, O. Bunau, M. Buongiorno Nardelli, M. Calandra, R. Car, C. Cavazzoni, D. Ceresoli, M. Cococcioni, N. Colonna, I. Carnimeo, A. Dal Corso, S. de Gironcoli, P. Delugas, R. A. DiStasio Jr., A. Ferretti, A. Floris, G. Fratesi, G. Fugallo, R. Gebauer, U. Gerstmann, F. Giustino, T. Gorni, J. Jia, M. Kawamura, H.-Y. Ko, A. Kokalj, E. Küçükbenli, M. Lazzeri, M. Marsili, N. Marzari, F. Mauri, N. L. Nguyen, H.-V. Nguyen, A. Otero-de-la-Roza, L. Paulatto, S. Poncé, D. Rocca, R. Sabatini, B. Santra, M. Schlipf, A. P. Seitsonen, A. Smogunov, I. Timrov, T. Thonhauser, P. Umari, N. Vast, X. Wu, and S. Baroni, *Advanced capabilities for materials modelling with Quantum ESPRESSO*, J. Phys.: Condens. Matter **29**, 465901 (2017).
